# Editorial: Metabolic Alterations in Neurodegenerative Disorders

**DOI:** 10.3389/fnagi.2022.833109

**Published:** 2022-02-18

**Authors:** Kristine K. Freude, Ines Moreno-Gonzalez, Carlos J. Rodriguez-Ortiz, David Baglietto-Vargas

**Affiliations:** ^1^Department of Veterinary and Animal Sciences, Faculty of Health and Medical Sciences, University of Copenhagen, Frederiksberg, Denmark; ^2^Departamento Biologia Celular, Genetica y Fisiologia, Instituto de Investigacion Biomedica de Malaga, Facultad de Ciencias, Universidad de Malaga, Malaga, Spain; ^3^Centro de Investigacion Biomedica en Red Sobre Enfermedades Neurodegenerativas, Madrid, Spain; ^4^Institute for Memory Impairments and Neurological Disorders, University of California, Irvine, Irvine, CA, United States

**Keywords:** neurodegenerative disorders, hyperlipidemia, metabolism, blood-brain barrier, astrocytes, ceramides, probiotics, anesthesia

The world's population is growing larger and older due to extended life span, resulting from improved medical intervention possibilities, assistance, and overall quality of life. However, associated with this increased life expectancy, the number of neurodegenerative disorders (NDDs) such as Alzheimer's disease (AD), Parkinson's disease (PD), Huntington's disease (HD), and many other diseases has increased exponentially in the past few decades, causing a progressive and irreversible deterioration of the brain and disturbing the daily activity of affected individuals and their families (Dugger and Dickson, 2017). Novel evidence has indicated that these NDDs are intimately connected to metabolic alterations, which facilitate or trigger the progression of these disorders (Muddapu et al., 2020). Therefore, this special issue focuses on investigating the factors and mechanisms by which metabolic alterations trigger the onset and enhance the progression of NDDs. As such, Hefner et al., highlighted the impact of metabolic alterations such as type 2 diabetes mellitus (T2DM), obesity and non-alcoholic fatty liver disease (NAFLD) into the development of different proteinopathies such as AD. Specifically, this review argues that cardiometabolic disorders are able to rise amyloid beta (Aβ) peptide levels in the periphery and subsequently cross the blood brain barrier and increase the Aβ levels in the brain. Furthermore, Li et al., demonstrate that T2DM increases iron concentration in several brain areas, causing neurotoxicity, which can lead to multiple neuronal diseases such as PD, AD, and HD. Overall, these studies highlight the importance of specific cardiometabolic diseases affecting both peripheral and central nervous system (CNS) through diverse mechanisms, triggering the onset, and progression of multiple NDDs.

One of the earliest pathological changes in many NDDs, including AD, affects cell metabolism and, more specifically, glucose metabolism in neurons (Gordon et al., 2018). In addition, emerging evidence supports the notion that glia cells play a profound role in stable brain metabolism and functionality of neurons. Consequently, studies investigating the glial contribution besides glucose metabolism could reveal functional insights into altered metabolic features in AD brains. In this special issue, Salcedo et al. investigate the role of branched-chain amino acids (BCAAs) in both primary astrocytes derived from familial AD (fAD) mouse models and from astrocytes derived from human induced pluripotent stem cells (hiPSCs). BCAA's are central to neurotransmitter cycling (Yudkoff et al., 1996) and the authors elegantly show that BCAA's are highly metabolized in astrocytes in order to synthesize glutamine. Moreover, they show that hiPSC-derived astrocytes carrying fAD mutations display a reduction in synthesis of neuroactive amino acids. These findings underline the importance of decreased alternative substrate usage in AD within the glia compartments of the brain and thereby, contribution to the overall hypometabolism present early on in AD pathology. Another interesting aspect presented in this special issue is the link between APOE status, abundance of ceramides and gender. Ceramides are central to sphingolipid metabolism and whilst reduced ceramide levels promote neuronal survival and fitness; an increase in ceramides, as observed in AD postmortem brains, leads to the opposite and damages neurons (Czubowicz and Strosznajder, 2014). Even though den Hoedt et al. showed limited associations between the APOE4 status and presences of long-chain ceramides [Cer(d18:1/24:0)], they were able to pinpoint another interesting correlation, which includes the observation that the female gender of the mice was affecting ceramide levels in a much stronger way than the APOE4 genotype. This study opens up a future venue of research, which could link specific metabolic alterations to the gender bias observed in AD, with females being twice as likely affected than males.

Another highly relevant study with focus on metabolic substrate and cognitive decline is the clinical study by Kreuzer et al., which identified brain metabolic asymmetric alterations as a common factor coupling neuronal degeneration and cognitive function. In addition, Tang et al. demonstrated that Gamma-Glutamyl transferase, a key enzyme used as indicator of potential hepatic or biliary illness, is commonly upregulated in obese women with mild cognitive decline. In general, these studies highlight the importance of metabolic alterations as a major causative factor leading to cognitive impairments in NDDs.

This collection also includes a review report by Fang et al., summarizing the critical role of Silent information regulator-1 (SIRT1) in the regulation of important biological processes in cellular homeostasis, such as cell growth, apoptosis, inflammation, differentiation, metabolism, and senescence. In regards to energy stress, the study highlights the relevant role of SIRT1 maintaining mitochondrial proper function and biogenesis. Similarly, Tyagi et al., demonstrate that hyperlipidemia, a hallmark characteristic of metabolic syndrome (MetS), may contribute to further alterations in metabolism, inflammation and damage of the blood-brain barrier (BBB) by lowering the level of SIRT3. Thus, these findings support the idea that the SIRT family of signaling proteins are important effectors of metabolic alterations driving several downstream pathological mechanisms resulting in major risks to develop NNDs.

Mitochondrial dysfunction and the formation of reactive oxygen species (ROS) are both common pathological mechanisms that trigger neurodegeneration in several dementia-associated diseases, including AD and PD (Buccellato et al., 2021). Therefore, interventions that mitigate these pathologies are often the focus of studies evaluating potential therapies for NDDs. In this regard, Nurrahma et al. performed a preclinical assay where rat models of PD were supplemented with probiotics in an effort to ameliorate energy metabolism impairments. They reported that rats treated with probiotic supplementation showed amelioration of motor deficits along with restored muscle mass. Additionally, treated animals displayed lower dopaminergic degeneration, elevated mitochondrial function and energy metabolism, and reduced PD pathology. Although ROS species formation is a major pathological event triggering AD and PD, little is known about this in HD. A novel study led by Villegas et al. discusses the importance of NADPH oxidases (NOXs) in neuronal cells and how NOX contributes to redox levels and affects important biological processes, such as neurogenesis, neurite outgrowth, and synaptic plasticity. These studies highlight ROS as a major harmful factor in NDDs, and how modulating NOX levels, as well as probiotic supplementation, could represent a novel therapeutic intervention to mitigate ROS.

General anesthetics is a common medical approach in many surgical procedure, however, recent clinical evidences have shown that patients under anesthesia can develop profound cognitive alterations, including post-operative delirium (POD) (Cottrell and Hartung, 2020). In regard with this, Lin et al. has showed that hip replacement, one of the most common medical procedure, is associated with clinical cognitive disorders. In addition, new scientific evidences showed that metabolic alterations and T2DM aggravate the risk to suffer POD (Hudetz et al., 2011). In this sense, Peng et al., have demonstrated that mice on a high fat diet (HFD) and under long-term exposure to the anesthetic isoflurane, developed significant insulin resistance (IR), and cognitive impairments. Metformin, an antidiabetic compound, could reverse the observed phenotypes of IR, tau hyperphosphorylation and cognitive deficits in mice. These studies demonstrate that general anesthetic procedure is a risk factor for development or progression of cognitive decline, which is aggravated when metabolic alterations are present in the patients.

Overall, this special issue contains a series of compelling studies that provide critical view into the mechanisms and pathological processes by which metabolic alterations drive the development of several NDDs.

## Author Contributions

KF, IM-G, CR-O, and DB-V have contributed to the manuscript writing and editing. DB-V has coordinated, reviewed, and submitted the editorial manuscript. All authors contributed to the article and approved the submitted version.

## Funding

This study was supported by awards from the Innovation Fund Denmark (BrainStem, 4108-00008B) (KF), Novo Nordisk Foundation (GliAD—NNF18OC0052369 and NNF19OC0058399) (KF), Spanish Ministry of Science and Innovation PID2019-108911RA-100 (DB-V) and PID2019-107090RA-100 (IM-G), Beatriz Galindo program BAGAL18/00052 (DB-V), RYC-2017-21879 (IM-G), UMA20-FEDERJA-104 (IM-G), and B1-2019_06 (IM-G).

## Conflict of Interest

The authors declare that the research was conducted in the absence of any commercial or financial relationships that could be construed as a potential conflict of interest.

## Publisher's Note

All claims expressed in this article are solely those of the authors and do not necessarily represent those of their affiliated organizations, or those of the publisher, the editors and the reviewers. Any product that may be evaluated in this article, or claim that may be made by its manufacturer, is not guaranteed or endorsed by the publisher.
